# Effectiveness of manual therapies: the UK evidence report

**DOI:** 10.1186/1746-1340-18-3

**Published:** 2010-02-25

**Authors:** Gert Bronfort, Mitch Haas, Roni Evans, Brent Leininger, Jay Triano

**Affiliations:** 1 Northwestern Health Sciences University, 2501 W 84th St, Bloomington, MN, USA; 2 University of Western States, 2900 NE 132nd Ave, Portland, OR, USA; 3 Canadian Memorial Chiropractic College, 6100 Leslie St, North York, ON, Canada; 4 McMaster University, 1280 Main St W, Hamilton, ON, Canada

## Abstract

**Background:**

The purpose of this report is to provide a succinct but comprehensive summary of the scientific evidence regarding the effectiveness of manual treatment for the management of a variety of musculoskeletal and non-musculoskeletal conditions.

**Methods:**

The conclusions are based on the results of systematic reviews of randomized clinical trials (RCTs), widely accepted and primarily UK and United States evidence-based clinical guidelines, plus the results of all RCTs not yet included in the first three categories. The strength/quality of the evidence regarding effectiveness was based on an adapted version of the grading system developed by the US Preventive Services Task Force and a study risk of bias assessment tool for the recent RCTs.

**Results:**

By September 2009, 26 categories of conditions were located containing RCT evidence for the use of manual therapy: 13 musculoskeletal conditions, four types of chronic headache and nine non-musculoskeletal conditions. We identified 49 recent relevant systematic reviews and 16 evidence-based clinical guidelines plus an additional 46 RCTs not yet included in systematic reviews and guidelines.

Additionally, brief references are made to other effective non-pharmacological, non-invasive physical treatments.

**Conclusions:**

Spinal manipulation/mobilization is effective in adults for: acute, subacute, and chronic low back pain; migraine and cervicogenic headache; cervicogenic dizziness; manipulation/mobilization is effective for several extremity joint conditions; and thoracic manipulation/mobilization is effective for acute/subacute neck pain. The evidence is inconclusive for cervical manipulation/mobilization alone for neck pain of any duration, and for manipulation/mobilization for mid back pain, sciatica, tension-type headache, coccydynia, temporomandibular joint disorders, fibromyalgia, premenstrual syndrome, and pneumonia in older adults. Spinal manipulation is not effective for asthma and dysmenorrhea when compared to sham manipulation, or for Stage 1 hypertension when added to an antihypertensive diet. In children, the evidence is inconclusive regarding the effectiveness for otitis media and enuresis, and it is not effective for infantile colic and asthma when compared to sham manipulation.

Massage is effective in adults for chronic low back pain and chronic neck pain. The evidence is inconclusive for knee osteoarthritis, fibromyalgia, myofascial pain syndrome, migraine headache, and premenstrual syndrome. In children, the evidence is inconclusive for asthma and infantile colic.

## Background

The impetus for this report stems from the media debate in the United Kingdom (UK) surrounding the scope of chiropractic care and claims regarding its effectiveness particularly for non-musculoskeletal conditions.

The domain of evidence synthesis is always embedded within the structure of societal values [1]. What constitutes evidence for specific claims is framed by the experience, knowledge, and standards of communities [2,3]. This varies substantially depending on jurisdictional restrictions by country and region. However, over the last several decades a strong international effort has been made to facilitate the systematic incorporation of standardized synthesized clinical research evidence into health care decision making [4].

### Evidence-Based Healthcare (EBH)

EBH is about doing the right things for the right people at the right time [5]. It does so by promoting the examination of best available clinical research evidence as the preferred process of decision making where higher quality evidence is available [6]. This reduces the emphasis on unsystematic clinical experience and pathophysiological rationale alone while increasing the likelihood of improving clinical outcomes [7]. The fact that randomized clinical trial (RCT) derived evidence of potentially effective interventions in population studies may not be translated in a straight forward manner to the management of individual cases is widely recognized [8-10]. However, RCTs comprise the body of information best able to meet existing standards for claims of benefit from care delivery. The evidence provided by RCTs constitutes the first line of recommended action for patients and contributes, along with informed patient preference, in guiding care [11]. Practice, as opposed to claims, is inherently interpretative within the context of patient values and ethical defensibility of recommendations [8,12]. Indeed, the need to communicate research evidence, or its absence, to patients for truly informed decision-making has become an important area of health care research and clinical practice [13,14].

While some may argue that EBH is more science than art [7], the skill required of clinicians to integrate research evidence, clinical observations, and patient circumstances and preferences is indeed artful [6]. It requires creative, yet informed improvisation and expertise to balance the different types of information and evidence, with each of the pieces playing a greater or lesser role depending on the individual patient and situation [15].

It has become generally accepted that providing evidence-based healthcare will result in better patient outcomes than non-evidence-based healthcare [7]. The debate of whether or not clinicians should embrace an evidence-based approach has become muted. Put simply by one author: "...anyone in medicine today who does not believe in it (EBH) is in the wrong business [7]." Many of the criticisms of EBH were rooted in confusion over what should be done when good evidence is available versus when evidence is weak or nonexistent. From this, misunderstandings and misperceptions arose, including concerns that EBH ignores patient values and preferences and promotes a cookbook approach [16]. When appropriately applied, EBH seeks to empower clinicians so they can develop fact-based independent views regarding healthcare claims and controversies. Importantly, it acknowledges the limitations of using scientific evidence alone to make decisions and emphasizes the importance of patients' values and preferences in clinical decision making [6].

The question is no longer "should" we embrace EBH but "how"? With EBH comes the need for new skills including: efficient literature search strategies and the application of formal rules of evidence in evaluating the clinical literature [6]. It is important to discern the role of the health care provider as an advisor who empowers informed patient decisions. This requires a healthy respect for which scientific literature to use and how to use it. "Cherry-picking" only those studies which support one's views or relying on study designs not appropriate for the question being asked does not promote doing the right thing for the right people at the right time.

Perhaps most critical is the clinician's willingness to change the way they practice when high quality scientific evidence becomes available. It requires flexibility born of intellectual honesty that recognizes one's current clinical practices may not *really *be in the best interests of the patient. In some cases this will require the abandonment of treatment and diagnostic approaches once believed to be helpful. In other cases it will require the acceptance and training in new methods. The ever-evolving scientific knowledge base demands that clinicians be accepting of the possibility that what is "right" today might not be "right" tomorrow. EBH requires that clinicians' actions are influenced by the evidence [17]. Importantly a willingness to change must accompany the ability to keep up to date with the constant barrage of emerging scientific evidence.

### Purpose

The purpose of this report is to provide a brief and succinct summary of the scientific evidence regarding the effectiveness of manual treatment as a therapeutic option for the management of a variety of musculoskeletal and non-musculoskeletal conditions based on the volume and quality of the evidence. Guidance in translating this evidence to application within clinical practice settings is presented.

## Methods

For the purpose of this report, manual treatment includes spinal and extremity joint manipulation or mobilization, massage and various soft tissue techniques. Manipulation/mobilization under anaesthesia was not included in the report due to the procedure's invasive nature. The conclusions of the report are based on the results of the most recent and most updated (spans the last five to ten years) systematic reviews of RCTs, widely accepted evidence-based clinical guidelines and/or technology assessment reports (primarily from the UK and US if available), and all RCTs not yet included in the first three categories. While critical appraisal of the included reviews and guidelines would be ideal, it is beyond the scope of the present report. The presence of discordance between the conclusions of systematic reviews is explored and described. The conclusions regarding effectiveness are based on comparisons with placebo controls (efficacy) or commonly used treatments which may or may not have been shown to be effective (relative effectiveness), as well as comparison to no treatment. The strength/quality of the evidence relating to the efficacy/effectiveness of manual treatment is graded according to an adapted version of the latest grading system developed by the US Preventive Services Task Force (see http://www.ahrq.gov/clinic/uspstf/grades.htm). The evidence grading system used for this report is a slight modification of the system used in the 2007 Joint Clinical Practice Guideline on low back pain from the American College of Physicians and the American Pain Society [18].

Through a search strategy using the databases MEDLINE (PubMed), Ovid, Mantis, Index to Chiropractic Literature, CINAHL, the specialized databases Cochrane Airways Group trial registry, Cochrane Complementary Medicine Field, and Cochrane Rehabilitation Field, systematic reviews and RCTs as well as evidence-based clinical guidelines were identified. Search restrictions were human subjects, English language, peer-reviewed and indexed journals, and publications before October 2009. In addition, we screened and hand searched reference citations located in the reviewed publications. The description of the search strategy is provided in Additional file 1 (Medline search strategy).

Although findings from studies using a nonrandomized design (for example observational studies, cohort studies, prospective clinical series and case reports) can yield important preliminary evidence, the primary purpose of this report is to summarize the results of studies designed to address efficacy, relative efficacy or relative effectiveness and therefore the evidence base was restricted to RCTs. Pilot RCTs not designed or powered to assess effectiveness, and RCTs designed to test the immediate effect of individual treatment sessions were not part of the evidence base in this report.

The quality of RCTs, which have not been formally quality-assessed within the context of systematic reviews or evidence based guidelines, was assessed by two reviewers with a scale assessing the risk of bias recommended for use in Cochrane systematic reviews of RCTs. Although the Cochrane Collaboration handbook http://www.cochrane.org/resources/handbook/ discourages that scoring be applied to the risk of bias tool, it does provide suggestion for how trials can be summarized. We have been guided by that suggestion and the adapted evidence grading system used in this report requires that we assess the validity and impact of the latest trial evidence. These additional trials are categorized as higher, moderate, or lower-quality as determined by their attributed risk of bias. For details, see Additional file 2 (The Cochrane Collaboration tool for assessing risk of bias and the rating of the bias for the purpose of this report).

The overall evidence grading system allows the strength of the evidence to be categorized into one of three categories: ***high quality evidence, moderate quality evidence, and inconclusive (low quality) evidence***. The operational definitions of these three categories follow below:

### High quality evidence

The available evidence usually includes consistent results from well-designed, well conducted studies in representative populations which assess the effects on health outcomes.

The evidence is based on at least two consistent higher-quality (*low risk of bias*) randomized trials. This conclusion is therefore unlikely to be strongly affected by the results of future studies.

### Moderate quality evidence

The available evidence is ***sufficient ***to determine the effectiveness relative to health outcomes, but confidence in the estimate is constrained by such factors as:

• The number, size, or quality of individual studies.

• Inconsistency of findings across individual studies.

• Limited generalizability of findings to routine practice.

• Lack of coherence in the chain of evidence.

The evidence is based on at least one higher-quality randomized trial (*low risk of bias*) with sufficient statistical power, two or more higher-quality (*low risk of bias*) randomized trials with some inconsistency; at least two consistent, lower-quality randomized trials (*moderate risk of bias*). As more information becomes available, the magnitude or direction of the observed effect could change, and this change may be large enough to alter the conclusion.

### Inconclusive (low quality) evidence

The available evidence is ***insufficient ***to determine effectiveness relative to health outcomes. Evidence is insufficient because of:

• The limited number or power of studies.

• Important flaws in study design or methods (*only high risk of bias studies available*).

• Unexplained inconsistency between higher-quality trials.

• Gaps in the chain of evidence.

• Findings not generalizable to routine practice.

• Lack of information on important health outcomes

For the purpose of this report a determination was made whether the inconclusive evidence appears favorable or non-favorable or if a direction could even be established (unclear evidence).

Additionally, brief evidence statements are made regarding other non-pharmacological, non-invasive physical treatments (for example exercise) and patient educational interventions, shown to be effective and which can be incorporated into evidence-based therapeutic management or co-management strategies in chiropractic practices. These statements are based on conclusions of the most recent and most updated (within last five to ten years) systematic reviews of randomized clinical trials and widely accepted evidence-based clinical guidelines (primarily from the UK and US if available) identified through our search strategy.

### Translating Evidence to Action

Translating evidence requires the communication of salient take-home messages in context of the user's applications [3]. There are two message applications for information derived from this work. First, the criteria for sufficiency of evidence differ depending on the context of the considered actions [8,19]. Sufficient evidence to proffer claims of effectiveness is defined within the socio-political context [20] of ethics and regulation. Separate is the second application of evidence to inform decision making for individual patients. Where there is strength of evidence and the risk of bias is small, the preferred choices require little clinical judgment. Alternatively, when evidence is uncertain and/or there is higher risk of bias, then greater emphasis is placed on the patient as an active participant [11]. This requires the clinician to effectively communicate research evidence to patients while assisting their informed decision-making [19].

**Figure 1:**
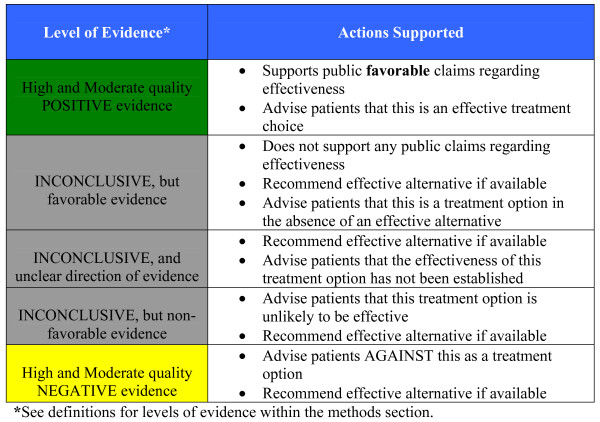
**Translating Evidence to Action**.

In summary, the information derived within this report are directed to two applications 1) the determination of supportable public claims of treatment effectiveness for chiropractic care within the context of social values; and 2) the use of evidence information as a basis for individualized health care recommendations using the hierarchy of evidence (Figure 1).

## Results

**Figure 2:**
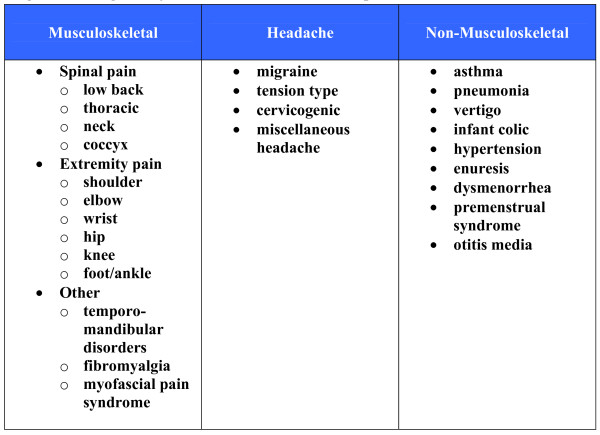
**Categories of Conditions included in this report**.

**Figure 3:**
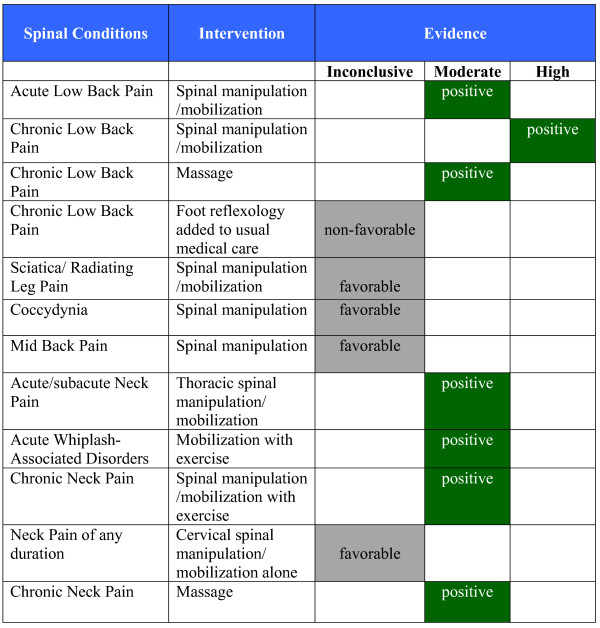
**Evidence Summary - Adults - Spinal Conditions**.

**Figure 4:**
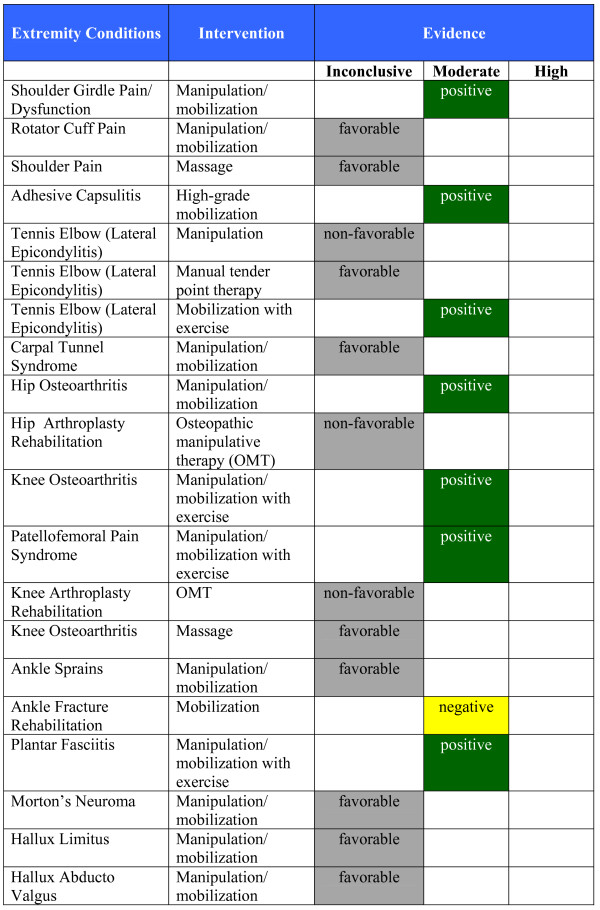
**Evidence Summary - Adults - Extremity Conditions**.

**Figure 5:**
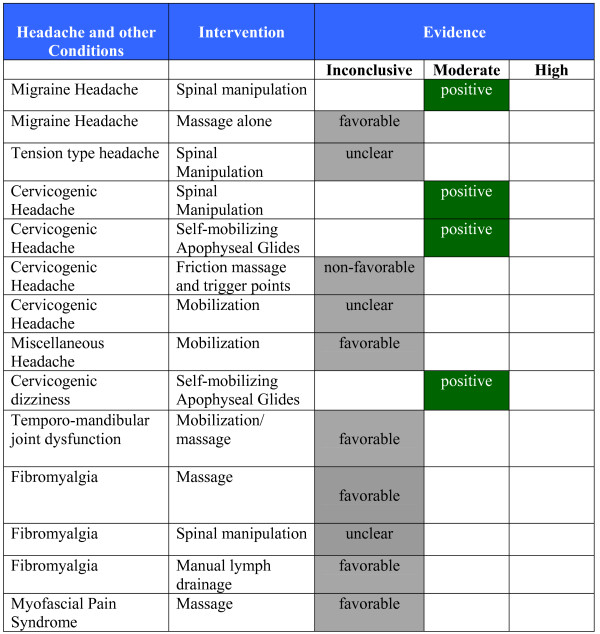
**Evidence Summary - Adults - Headache and Other Conditions**.

**Figure 6:**
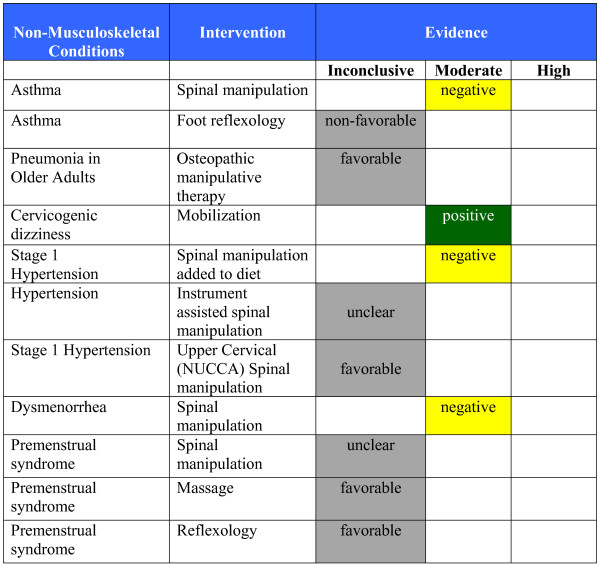
**Evidence Summary - Adults - Non-Musculoskeletal Conditions**.

**Figure 7:**
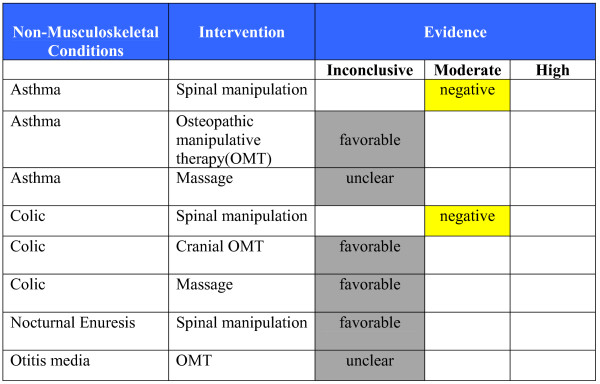
**Evidence Summary - Pediatrics - Non-Musculoskeletal Conditions**.

By September 2009, 26 categories of conditions were located containing RCT evidence for the use of manual therapy: 13 musculoskeletal conditions, four types of chronic headache and nine non-musculoskeletal conditions (Figure 2). We identified 49 recent relevant systematic reviews and 16 evidence-based clinical guidelines plus an additional 46 RCTs not yet included within the identified systematic reviews and guidelines. A number of other non-invasive physical treatments and patient education with evidence of effectiveness were identified including exercise, yoga, orthoses, braces, acupuncture, heat, electromagnetic field therapy, TENS, laser therapy, cognitive behavioral therapy and relaxation. The report presents the evidence of effectiveness or ineffectiveness of manual therapy as evidence summary statements at the end of the section for each condition and in briefer summary form in Figures 3, 4, 5, 6, and 7. Additionally, definitions and brief diagnostic criteria for the conditions reviewed are provided. Diagnostic imaging for many conditions is indicated by the presence of "red flags" suggestive of serious pathology. Red flags may vary depending on the condition under consideration, but typically include fractures, trauma, metabolic disorders, infection, metastatic disease, and other pathological disease processes contraindicative to manual therapy.

### Non-specific Low Back Pain (LBP)

#### Definition

Non-specific LBP is defined as soreness, tension, and/or stiffness in the lower back region for which it is not possible to identify a specific cause of pain [21].

#### Diagnosis

Diagnosis of non-specific LBP is derived from the patient's history with an unremarkable neurological exam and no indicators of potentially serious pathology. Imaging is only indicated in patients with a positive neurological exam or presence of a "red flag" [21-24].

### Evidence base for manual treatment

#### Systematic reviews (most recent)

Since 2004, five systematic reviews made a comprehensive evaluation of the benefit of spinal manipulation for non-specific LBP [25-30]. Approximately 70 RCTs were summarized. The reviews found that spinal manipulation was superior to sham intervention and similar in effect to other commonly used efficacious therapies such as usual care, exercise, or back school. For sciatica/radiating leg pain, three reviews [18,25,27] found manipulation to have limited evidence. Furlan et al [30] concluded massage is beneficial for patients with subacute and chronic non-specific low-back pain based on a review of 13 RCTs.

#### Evidence-based clinical guidelines

Since 2006, four guidelines make recommendations regarding the benefits of manual therapies for the care of LBP: NICE [21,31], The American College of Physicians/American Pain Society [18,22], European guidelines for chronic LBP [23], and European guidelines for acute LBP [24]. The number of RCTs included within the various guidelines varied considerably based on their scope, with the NICE guidelines including eight trials and The American College of Physicians/American Pain Society guidelines including approximately 70 trials. These guidelines in aggregate recommend spinal manipulation/mobilization as an effective treatment for acute, subacute, and chronic LBP. Massage is also recommended for the treatment of subacute and chronic LBP.

#### Recent randomized clinical trials not included in above

Hallegraeff et al [32] compared a regimen of spinal manipulation plus standard physical therapy to standard physical therapy for acute LBP. Overall there were no differences between groups for pain and disability post treatment. Prediction rules may have affected outcomes. This study had a high risk of bias.

Rasmussen et al [33] found patients receiving extension exercise or receiving extension exercise plus spinal manipulation experienced a decrease in chronic LBP, but no differences were noted between groups. This study had a high risk of bias.

Little et al [34] found Alexander technique, exercise, and massage were all superior to control (normal care) at three months for chronic LBP and disability. This study had a moderate risk of bias.

Wilkey et al [35] found chiropractic management was superior to NHS pain clinic management for chronic LBP at eight weeks for pain and disability outcomes. This study had a high risk of bias.

Bogefeldt et al [36] found manual therapy plus advice to stay active was more effective than advice to stay active alone for reducing sick leave and improving return to work at 10 weeks for acute LBP. No differences between the groups were noted at two years. This study had a low risk of bias.

Hancock et al [37] found spinal mobilization in addition to medical care was no more effective than medical care alone at reducing the number of days until full recovery for acute LBP. This study had a low risk of bias.

Ferreira et al [38] found spinal manipulation was superior to general exercise for function and perceived effect at eight weeks in chronic LBP patients, but no differences were noted between groups at six and 12 months. This study had a moderate risk of bias.

Eisenberg et al [39] found that choice of complementary therapies (including chiropractic care) in addition to usual care was no different from usual care in bothersomeness and disability for care of acute LBP. The trial did not report findings for any individual manual therapy. This study had a low risk of bias.

Hondras et al [40] found lumbar flexion-distraction was superior to minimal medical care at 3,6,9,12, and 24 weeks for disability related to subacute or chronic LBP, but spinal manipulation was superior to minimal medical care only at three weeks. No differences between spinal manipulation and flexion-distraction were noted for any reported outcomes. Global perceived improvement was superior at 12 and 24 weeks for both manual therapies compared to minimal medical care. This study had a low risk of bias.

Mohseni-Bandpei et al [41] showed that patients receiving manipulation/exercise for chronic LBP reported greater improvement compared with those receiving ultrasound/exercise at both the end of the treatment period and at 6-month follow-up. The study had a high risk of bias.

Beyerman et al [42] evaluated the efficacy of chiropractic spinal manipulation, manual flexion/distraction, and hot pack application for the treatment of LBP of mixed duration from osteoarthritis (OA) compared with moist heat alone. The spinal manipulation group reported more and faster short term improvement in pain and range of motion. The study had a high risk of bias.

Poole et al [43] showed that adding either foot reflexology or relaxation training to usual medical care in patients with chronic LBP is no more effective than usual medical care alone in either the short or long term. The study had a moderate risk of bias.

Zaproudina et al [44] found no differences between groups (bonesetting versus exercise plus massage) at one month or one year for pain or disability. The global assessment score of improvement was superior for the bonesetting group at one month. This study had a high risk of bias.

#### Evidence Summary (See Figure 3)

◦ High quality evidence that spinal manipulation/mobilization is an effective treatment option for subacute and chronic LBP in adults [18,21,23].

◦ Moderate quality evidence that spinal manipulation/mobilization is an effective treatment option for subacute and chronic LBP in older adults [40].

◦ Moderate quality evidence that spinal manipulation/mobilization is an effective treatment option for acute LBP in adults [18,24].

◦ Moderate evidence that adding spinal mobilization to medical care does not improve outcomes for acute LBP in adults [37].

◦ Moderate quality evidence that massage is an effective treatment for subacute and chronic LBP in adults [22,30].

◦ Inconclusive evidence in a favorable direction regarding the use of manipulation for sciatica/radiating leg pain [22,25,27].

◦ Inconclusive evidence in a non-favorable direction regarding the addition of foot reflexology to usual medical care for chronic LBP [43].

#### Other effective non-invasive physical treatments or patient education

Advice to stay active, interdisciplinary rehabilitation, exercise therapy, acupuncture, yoga, cognitive-behavioral therapy, or progressive relaxation for chronic LBP and superficial heat for acute LBP [18,22].

### Non-specific mid back pain

#### Definition

Non-specific thoracic spine pain is defined as soreness, tension, and/or stiffness in the thoracic spine region for which it is not possible to identify a specific cause of pain [45].

#### Diagnosis

Diagnosis of non-specific thoracic spine pain is derived from the patient's history with an unremarkable neurological exam and no indicators of potentially serious pathology. Imaging is only indicated in patients with a positive neurological exam or presence of a "red flag" [45,46].

### Evidence base for manual treatment

#### Systematic reviews (most recent)

No systematic reviews addressing the role of manual therapy in thoracic spine pain that included randomized clinical trials were located.

#### Evidence-based clinical guidelines

The Australian acute musculoskeletal pain guidelines group concludes there is evidence from one small pilot study [47] that spinal manipulation is effective compared to placebo for thoracic spine pain.

#### Recent randomized clinical trials not included in above

Multiple randomized clinical trials investigating the use of thoracic spinal manipulation were located [48-53]; however, most of the trials assessed the effectiveness of thoracic manipulation for neck or shoulder pain.

#### Evidence Summary (See Figure 3)

◦ Inconclusive evidence in a favorable direction regarding the use of spinal manipulation for mid back pain [47].

#### Other effective non-invasive physical treatments or patient education

None

### Mechanical neck pain

#### Definition

Mechanical neck pain is defined as pain in the anatomic region of the neck for which it is not possible to identify a specific pathological cause of pain [54,55]. It generally includes neck pain, with or without pain in the upper limbs which may or may not interfere with activities of daily living (Grades I and II). Signs and symptoms indicating significant neurologic compromise (Grade III) or major structural pathology (Grade IV including fracture, vertebral dislocation, neoplasm, etc.) are NOT included.

#### Diagnosis

Diagnosis of mechanical neck pain is derived from the patient's history. Imaging is only indicated in patients with a positive neurological exam or presence of a "red flag" [54,56].

### Evidence base for manual treatment

#### Systematic reviews (most recent)

The recently published best evidence synthesis by the Bone and Joint Decade 2000-2010 Task Force on Neck Pain and Its Associated Disorders represents the most recent and comprehensive systematic review of the literature for non-invasive interventions, including manual treatment, for neck pain [55]. For whiplash associated disorders, they concluded that mobilization and exercises appear more beneficial than usual care or physical modalities. For Grades I and II neck pain, they concluded that the evidence suggests that manual treatment (including manipulation and mobilization) and exercise interventions, low-level laser therapy and perhaps acupuncture are more effective than no treatment, sham or alternative interventions. No one type of treatment was found to be clearly superior to any other. They also note that manipulation and mobilization yield comparable results. Conclusions regarding massage could not be made due to lack of evidence.

Since 2003, there were five other systematic reviews [29,57-60]. One found that spinal manipulation was effective for non-specific neck pain alone and in combination with exercise [29], while two found effectiveness only for the combination of spinal manipulation and exercise [58,60]. Differences between review conclusions are expected. It is likely they can be attributed to additional primary studies and diversity in review strategies, including inclusion criteria, methodological quality scoring, and evidence determination.

#### Evidence-based clinical guidelines

The American Physical Therapy Association's guidelines on neck pain recommends utilizing cervical manipulation and mobilization procedures to reduce neck pain based on strong evidence [56]. They found cervical manipulation and mobilization with exercise to be more effective for reducing neck pain and disability than manipulation and mobilization alone. Thoracic spine manipulation is also recommended for reducing pain and disability in patients with neck and neck-related arm pain based on weak evidence.

#### Recent randomized clinical trials not included in above

Häkkinen et al used a cross-over design to compare manual therapy and stretching for chronic neck pain [61]. Manual therapy was more effective than stretching at four weeks, but no difference between the two therapies was noted at 12 weeks. This study had a high risk of bias.

González-Iglesias et al examined the effectiveness of adding general thoracic spine manipulation to electrotherapy/thermal therapy for acute neck pain. In two separate trials they found an advantage for the manipulation group in terms of pain and disability [62,63]. The trials had moderate to low risk of bias.

Walker et al compared manual therapy with exercise to advice to stay active and placebo ultrasound [64]. The manual therapy group reported less pain (in the short term) and more improvement and less disability (in the long term) than the placebo group. This study had a low risk of bias.

Cleland et al [65] showed that thoracic spine thrust mobilization/manipulation results in a significantly greater short-term reduction in pain and disability than does thoracic non-thrust mobilization/manipulation in people with mostly subacute neck pain. The study had a low risk of bias.

Fernandez et al [66] found that adding thoracic manipulation to a physical therapy program was effective in treating neck pain due to whiplash injury. The study had a high risk of bias.

Savolainen et al [49] compared the effectiveness of thoracic manipulations with instructions for physiotherapeutic exercises for the treatment of neck pain in occupational health care. The effect of the manipulations was more favorable than the personal exercise program in treating the more intense phase of pain. The study had a moderate risk of bias.

Zaproudina et al [67] assessed the effectiveness of traditional bone setting (mobilization) of joints of extremities and the spine for chronic neck pain compared with conventional physiotherapy or massage. The traditional bone setting was superior to the other two treatments in both in the short and long term. The study had a moderate risk of bias.

Sherman et al compared massage therapy to self-care for chronic neck pain. Massage was superior to self-care at 4 weeks for both neck disability and pain [68]. A greater proportion of massage patients reported a clinically significant improvement in disability than self-care patients at four weeks, and more massage patients reported a clinically significant improvement in pain at four and 10 weeks. No statistically significant differences between groups were noted at 26 weeks. This study had a low risk of bias.

#### Evidence Summary (See Figure 3)

◦ Moderate quality evidence that mobilization combined with exercise is effective for acute whiplash-associated disorders [55].

◦ Moderate quality evidence that spinal manipulation/mobilization combined with exercise is effective for chronic non-specific neck pain [55,58].

◦ Moderate quality evidence that thoracic spinal manipulation/mobilization is effective for acute/subacute non-specific neck pain [62,63,65,66].

◦ Moderate quality evidence that spinal manipulation is similar to mobilization for chronic non-specific neck pain [55,58].

◦ Moderate quality evidence that massage therapy is effective for non-specific chronic neck pain [68].

◦ Inconclusive evidence in a favorable direction for cervical spinal manipulation/mobilization alone for neck pain of any duration [29,55,58].

#### Other effective non-invasive physical treatments or patient education

Exercise, low-level laser therapy, acupuncture [55]

### Coccydynia

#### Definition

Coccydynia is defined as symptoms of pain in the region of the coccyx [69].

#### Diagnosis

Diagnosis of coccydynia is derived from the patient's history and exam with no indicators of potentially serious pathology. Imaging is only indicated in patients with a presence of a "red flag" [46,69].

### Evidence base for manual treatment

#### Systematic reviews (most recent)

None located

#### Evidence-based clinical guidelines

None located

#### Recent randomized clinical trials not included in above

Maigne et al [70] found manipulation was more effective than placebo for pain relief and disability in the treatment of coccydynia at one month. This study had a moderate risk of bias.

#### Evidence Summary (See Figure 3)

◦ Inconclusive evidence in a favorable direction for the use of spinal manipulation in the treatment of coccydynia [70].

#### Other effective non-invasive physical treatments or patient education

None

### Shoulder pain

#### Definition

Shoulder pain is defined as soreness, tension, and/or stiffness in the anatomical region of the shoulder and can be secondary to multiple conditions including, but not limited to rotator cuff disease and adhesive capsulitis.

#### Diagnosis

Diagnosis of shoulder pain is derived mainly from the patient's history and physical exam with no indicators of potentially serious pathology. Imaging studies are confirmatory for diagnoses of rotator cuff disorders, osteoarthritis, glenohumeral instability, and other pathologic causes of shoulder pain [71].

### Evidence base for manual treatment

#### Systematic reviews (most recent)

Two systematic reviews evaluated the benefit of manual therapy for shoulder pain [72,73]. Six RCTs evaluating the effectiveness of manual therapy for the treatment of shoulder pain were included [74-79]. Five of the trials evaluated mobilization [74-77,79] while one trial evaluated the use of manipulation and mobilization [78] for shoulder pain. The review concluded there is weak evidence that mobilization added benefit to exercise for rotator cuff disease.

#### Evidence-based clinical guidelines

The Philadelphia Panel's evidence based clinical practice guidelines on selected rehabilitation interventions for shoulder pain concluded there is insufficient evidence regarding the use of therapeutic massage for shoulder pain [80].

#### Recent randomized clinical trials not included in above

Vermeulen et al [81] found that high-grade mobilization techniques were more effective than low-grade mobilization techniques for active range of motion (ROM), passive ROM, and shoulder disability for adhesive capsulitis at three to 12 months. No differences were noted for pain or mental and physical general health. Both groups showed improvement in all outcome measures. This study had low risk of bias.

van den Dolder and Roberts [82] found massage was more effective than no treatment for pain, function, and ROM over a two week period in patients with shoulder pain. This study had moderate risk of bias.

Bergman et al [51] found no differences between groups during the treatment period (6 wks). More patients reported being "recovered" in the usual care plus manipulative/mobilization group at 12 and 52 weeks compared to usual care alone. This study had low risk of bias.

Johnson et al [83] found no differences in pain or disability between anterior and posterior mobilization for the care of adhesive capsulitis. This study had a high risk of bias.

Guler-Uysal et al [84] concluded that deep friction massage and mobilization exercises was superior in the short term to physical therapy including diathermy for adhesive capsulitis. The study had a high risk of bias.

#### Evidence Summary (See Figure 4)

◦ Moderate quality evidence that high-grade mobilization is superior to low-grade mobilization for reduction of disability, but not for pain, in adhesive capsulitis [81].

◦ Inconclusive evidence in an unclear direction for a comparison of anterior and posterior mobilization for adhesive capsulitis [83].

◦ Moderate evidence favors the addition of manipulative/mobilization to medical care for shoulder girdle pain and dysfunction [51].

◦ Inconclusive evidence in a favorable direction for massage in the treatment of shoulder pain [82].

◦ Inconclusive evidence in a favorable direction for mobilization/manipulation in the treatment of rotator cuff pain [72].

#### Other effective non-invasive physical treatments or patient education

Exercise therapy [80]

### Lateral epicondylitis

#### Definition

Lateral epicondylitis is defined as pain in the region of the lateral epicondyle which is exacerbated by active and resistive movements of the extensor muscles of the forearm [85].

#### Diagnosis

Diagnosis is made solely from the patient's history and clinical examination [71].

### Evidence base for manual treatment

#### Systematic reviews (most recent)

Three systematic reviews evaluating the benefit of manual therapy for lateral epicondylitis have been identified [86-88]. Eight RCTs were included [89-96] in the systematic reviews examining the effect of various manual therapies including elbow [89] and wrist manipulation [92], cervical spine [90] and elbow mobilization [91,93,95], and cross-friction massage [94-96]. Bisset et al [86] concluded there is some evidence of positive initial effects of manual techniques (massage/mobilization) for lateral epicondylitis, but no long term evidence. Smidt et al [88] concluded there is insufficient evidence to draw conclusions on the effectiveness of mobilization techniques for lateral epicondylitis.

#### Evidence-based clinical guidelines

None located

#### Recent randomized clinical trials not included in above

Verhaar et al [97] showed that corticosteroid injection was superior to Cyriax physiotherapy for the number of pain free subjects at six weeks. No differences between groups were noted at one year. This study had a high risk of bias.

Bisset et al [98] found corticosteroid injections were superior to elbow mobilization with exercise which was superior to wait and see approaches for pain-free grip strength, pain intensity, function, and global improvement at six weeks. However, both elbow mobilization with exercise and the wait and see approach were superior to corticosteroid injections at six months and one year for all of the previously reported outcomes. This study had a low risk of bias.

Nourbakhsh and Fearon [99] found oscillating energy manual therapy (tender point massage) was superior to placebo manual therapy for pain intensity and function. This study had a high risk of bias due to sample size (low risk of bias otherwise).

#### Evidence Summary (See Figure 4)

◦ Moderate quality evidence that elbow mobilization with exercise is inferior to corticosteroid injections in the short term and superior in the long term for lateral epicondylitis [98].

◦ Inconclusive evidence in a favorable direction regarding the use of manual oscillating tender point therapy of the elbow for lateral epicondylitis [99].

#### Other effective non-invasive physical treatments or patient education

Laser therapy, acupuncture [86,100,101]

### Carpal tunnel syndrome

#### Definition

Carpal tunnel syndrome is defined as compression of the median nerve as it passes through the carpal tunnel in the wrist [102].

#### Diagnosis

Diagnosis of carpal tunnel syndrome is made from the patient's history, physical exam, and confirmatory electrodiagnostic tests [102].

### Evidence base for manual treatment

#### Systematic reviews (most recent)

Since 2003, four systematic reviews evaluated the benefit of manual therapy for carpal tunnel syndrome [87,103-105]. Two RCTs evaluating the effectiveness of manual therapy were included [106,107]. One of the trials examined the use of spinal and upper extremity manipulation [106], while the other trial examined the use of wrist manipulation [107] for carpal tunnel syndrome. The reviews concluded uncertain or limited evidence for manipulation/mobilization.

#### Evidence-based clinical guidelines

The American Academy of Orthopaedic Surgeons clinical practice guideline on the treatment of carpal tunnel syndrome [102] made no recommendations for or against the use of manipulation or massage therapy due to insufficient evidence.

#### Recent randomized clinical trials not included in above

None

#### Evidence Summary (See Figure 4)

◦ Inconclusive evidence in a favorable direction for manipulation/mobilization in the treatment of carpal tunnel syndrome [87,103,105].

#### Other effective non-invasive physical treatments or patient education

Splinting [102]

### Hip pain

#### Definition

Hip pain is defined as soreness, tension, and/or stiffness in the anatomical region of the hip and can be secondary to multiple conditions including hip osteoarthritis.

#### Diagnosis

Diagnosis of hip pain is derived from the patient's history and physical exam with an unremarkable neurological exam and no indicators of potentially serious pathology. Imaging studies are confirmatory for diagnoses of moderate or severe osteoarthritis [108,109].

### Evidence base for manual treatment

#### Systematic reviews (most recent)

One systematic review evaluating manual therapy for hip pain has been published [110]. One RCT evaluating the effectiveness of hip manipulation for the treatment of hip osteoarthritis was included in the published systematic review [111]. The review concluded there is limited evidence for manipulative therapy combined with multimodal or exercise therapy for hip osteoarthritis.

#### Evidence-based clinical guidelines

The NICE national clinical guidelines for care and management of adults with osteoarthritis [112] recommends manipulation and stretching should be considered as an adjunct to core treatment, particularly for osteoarthritis of the hip. This recommendation is based on the results of one RCT.

The orthopaedic section of the American Physical Therapy Association's guidelines on hip pain and mobility deficits [108] recommends clinicians should consider the use of manual therapy procedures to provide short-term pain relief and improve hip mobility and function in patients with mild hip osteoarthritis based on moderate evidence.

#### Recent randomized clinical trials not included in above

Licciardone et al found decreased rehabilitation efficiency with osteopathic manipulative therapy (OMT) compared to sham OMT following hip arthroplasty. No other significant differences were found between the two groups [113]. This study had a high risk of bias.

#### Evidence Summary (See Figure 4)

◦ Moderate quality evidence that hip manipulation is superior to exercise for the treatment of the symptoms of hip osteoarthritis [111].

◦ Inconclusive evidence in a non-favorable direction regarding osteopathic manipulative therapy for rehabilitation following total hip arthroplasty [113].

#### Other effective non-invasive physical treatments or patient education

Exercise therapy, advice about weight loss, and appropriate footwear [108,112,114]

### Knee pain

#### Definition

Knee pain is defined as soreness, tension, and/or stiffness in the anatomical region of the knee and can be secondary to multiple conditions including knee osteoarthritis or patellofemoral pain syndrome.

#### Diagnosis

Diagnosis of knee pain is derived from the patient's history and physical exam with an unremarkable neurological exam and no indicators of potentially serious pathology. Imaging studies are confirmatory for diagnoses of moderate or severe osteoarthritis [109,112].

### Evidence base for manual treatment

#### Systematic reviews (most recent)

As of September 2009, one systematic review evaluating the benefit of manual therapy for knee pain has been identified [110]. Ten RCT's evaluating the effectiveness of manual therapy for the treatment of knee pain were included in the published systematic review [115-124]. Both osteoarthritis knee pain and patellofemoral pain syndrome were included in the conditions reviewed. Various manual therapy techniques including spinal mobilization [115,116,119], spinal manipulation [118,123], knee mobilization [115-117,120-124], and knee manipulation [121] were examined within the review. The review concludes there is fair evidence for manipulative therapy of the knee and/or full kinetic chain (Sacro-iliac to foot), combined with multimodal or exercise therapy for knee osteoarthritis and patellofemoral pain syndrome.

#### Evidence-based clinical guidelines

The NICE national clinical guidelines for care and management of adults with osteoarthritis [112] recommends manipulation and stretching should be considered as an adjunct to core treatment.

#### Recent randomized clinical trials not included in above

Pollard et al [125] assessed a manual therapy protocol compared to non-forceful manual contact (control). They concluded that a short term of manual therapy significantly reduced pain compared to the control group. This study had a high risk of bias.

Perlman et al [126] found massage therapy was more effective than wait list control for osteoarthritis related knee pain, stiffness, and function. This study had a high risk of bias.

Licciardone et al [113] assessed osteopathic manipulative treatment following knee arthroplasty. This study found decreased rehabilitation efficiency with OMT compared to sham OMT; otherwise, no significant differences were found between the two groups. This study had a high risk of bias.

#### Evidence Summary (See Figure 4)

◦ Moderate quality evidence that manual therapy of the knee and/or full kinetic chain (SI to foot) combined with multimodal or exercise therapy is effective for the symptoms of knee osteoarthritis [110].

◦ Moderate quality evidence that manual therapy of the knee and/or full kinetic chain (SI to foot) combined with multimodal or exercise therapy is effective for patellofemoral pain syndrome [110].

◦ Inconclusive evidence in a favorable direction that massage therapy is effective for the symptoms of knee osteoarthritis [126].

◦ Inconclusive evidence in a non-favorable direction for the effectiveness of osteopathic manipulative therapy for rehabilitation following total hip or knee arthroplasty [113].

#### Other effective non-invasive physical treatments or patient education

Exercise therapy, advice about weight loss, appropriate footwear, pulsed electromagnetic field therapy, acupuncture, and TENS [112,127-131]

### Ankle and foot conditions

#### Definition

A variety of conditions are included under ankle and foot conditions including ankle sprains, plantar fasciitis, morton's neuroma, hallux limitus/rigidus, and hallux abducto valgus.

#### Diagnosis

The diagnosis of ankle/foot conditions relies mainly on the patient's history and physical examination. Imaging studies are indicated for morton's neuroma or in the presence of potential pathology [109].

### Evidence base for manual treatment

#### Systematic reviews (most recent)

As of September 2009, two systematic reviews evaluating the benefit of manual therapy for ankle and foot conditions have been published [110,132]. The ankle and foot conditions reviewed included ankle sprain, plantar fasciitis, morton's neuroma, hallux limitus, and hallux abducto valgus. Thirteen RCTs evaluating the effectiveness of manual therapy for the treatment of various ankle and foot conditions were included in the published systematic reviews [133-145]. Of the thirteen trials, six examined the use of ankle/foot manipulation [134,136,137,139-141], six examined the use of ankle/foot mobilization [133,135,138,143-145], and one trial examined the combined use of manipulation and mobilization [142].

The review by Brantingham et al concluded there is fair evidence for manipulative therapy of the ankle and/or foot combined with multimodal or exercise therapy for ankle inversion sprain [110]. The same authors found limited evidence for manipulative therapy combined with multimodal or exercise therapy for plantar fasciitis, metatarsalgia, and hallux limitus and insufficient evidence for the use of manual therapy for hallux abducto valgus.

The review by van der Wees et al concluded it is likely that manual mobilization has an initial effect on dorsiflexion range of motion after ankle sprains [132].

#### Evidence-based clinical guidelines

None making recommendations based on RCTs were located

#### Recent randomized clinical trials not included in above

Wynne et al found an osteopathic manipulative therapy group had greater improvement in plantar fasciitis symptoms versus placebo control. This study had a high risk of bias [146].

Cleland et al compared manual therapy with exercise to electrotherapy with exercise for patients with plantar heel pain [147]. They found manual therapy plus exercise was superior. This study had a low risk of bias.

Lin et al found the addition of manual therapy (mobilization) to a standard physiotherapy program provided no additional benefit compared to the standard physiotherapy program alone for rehabilitation following ankle fracture [148]. This study had a low risk of bias.

#### Evidence Summary (See Figure 4)

◦ Moderate quality evidence that mobilization is of no additional benefit to exercise in the rehabilitation following ankle fractures [148].

◦ Moderate quality evidence that manual therapy of the foot and/or full kinetic chain (SI to foot) combined with exercise therapy is effective for plantar fasciitis [147].

◦ Inconclusive evidence in a favorable direction for the effectiveness of manual therapy with multimodal or exercise therapy for ankle sprains [110].

◦ Inconclusive evidence in a favorable direction regarding the effectiveness of manual therapy for morton's neuroma, hallux limitus, and hallux abducto valgus [110].

#### Other effective non-invasive physical treatments or patient education

Stretching and foot orthoses for plantar fasciitis [149], ankle supports for ankle sprains [150]

### Temporomandibular disorders

#### Definition

Temporomandibular disorders consist of a group of pathologies affecting the masticatory muscles, temporomandibular joint, and related structures [151].

#### Diagnosis

Diagnosis of temporomandibular disorders is derived from the patient's history and physical exam with no indicators of potentially serious pathology [151,152].

### Evidence base for manual treatment

#### Systematic reviews (most recent)

As of September 2009, two systematic reviews evaluating the benefit of manual therapy for temporomandibular dysfunction have been published [153,154]. Three RCTs evaluating the effectiveness of manual therapy were included in the published systematic reviews [155-157]. Two of the trials examined the effectiveness of mobilization [155,156] and one trial assessed massage [157]. The reviews conclude there is limited evidence for the use of manual therapy in the treatment of temporomandibular dysfunction.

#### Evidence-based clinical guidelines

None located

#### Recent randomized clinical trials not included in above

Monaco et al [158] examined the effects of osteopathic manipulative treatment on mandibular kinetics compared to a no treatment control group; however, no between group analysis was performed. This study had a high risk of bias.

Ismail et al [159] found physical therapy including mobilization in addition to splint therapy was superior to splint therapy alone after three months of treatment for active mouth opening. No differences were found between groups for pain. This study had a moderate risk of bias.

#### Evidence Summary (See Figure 5)

◦ Inconclusive evidence in a favorable direction regarding mobilization and massage for temporomandibular dysfunction [154].

#### Other effective non-invasive physical treatments or patient education

None

### Fibromyalgia

#### Definition

Fibromyalgia syndrome (FMS) is a common rheumatological condition characterized by chronic widespread pain and reduced pain threshold, with hyperalgesia and allodynia [160].

#### Diagnosis

Diagnosis of fibromyalgia is made primarily from the patient's history and physical exam. The American College of Rheumatology have produced classification criteria for fibromyalgia including widespread pain involving both sides of the body, above and below the waist for at least three months and the presence of 11 out of 18 possible pre-specified tender points [161].

### Evidence base for manual treatment

#### Systematic reviews (most recent)

Since 2004, three systematic reviews evaluating the benefit of manual therapy for fibromyalgia have been published [162-164]. Six RCTs evaluating the effectiveness of manual therapy for the treatment of fibromyalgia were included in the published systematic reviews [165-170]. Five of the studies assessed the effectiveness of spinal manipulation for fibromyalgia [165-169], while one assessed the effectiveness of massage [170].

Schneider et al [162] conclude there is moderate level evidence from several RCTs and a systematic review [171] that massage is helpful in improving sleep and reducing anxiety in chronic pain; however, few of the studies included in the systematic review [162] specifically investigated fibromyalgia.

Ernst [163] states that the current trial evidence is insufficient to conclude that chiropractic is an effective treatment of fibromyalgia.

Goldenberg et al [164] conclude there is weak evidence of efficacy for chiropractic, manual, and massage therapy in the treatment of fibromyalgia.

#### Evidence-based clinical guidelines

The 2007 a multidisciplinary task force with members from 11 European countries published evidence based recommendation for FMS [160]. The task force notes the clinical trial evidence for manual therapy is lacking.

#### Randomized clinical trials not included in above

Ekici et al [172] found improvement was higher in the manual lymph drainage group compared to connective tissue massage on the fibromyalgia impact questionnaire, but no differences were noted between groups for pain, pain pressure threshold, or health related quality of life. This study had a moderate risk of bias.

#### Evidence Summary (See Figure 5)

◦ Inconclusive evidence in a favorable direction regarding the effectiveness of massage and manual lymph drainage for the treatment of fibromyalgia [162,172].

◦ Inconclusive evidence in an unclear direction regarding the effectiveness of spinal manipulation for the treatment of fibromyalgia [162].

#### Other effective non-invasive physical treatments or patient education

Heated pool treatment with or without exercise, supervised aerobic exercise [160,173]

### Myofascial Pain Syndrome

#### Definition

Myofascial pain syndrome is a poorly defined condition that requires the presence of myofascial trigger points.

#### Diagnosis

Diagnosis of myofascial pain syndrome is made exclusively from the patient's history and physical exam.

### Evidence base for manual treatment

#### Systematic reviews (most recent)

As of September 2009, one systematic review evaluating the benefit of manual therapy for myofascial pain syndrome was identified, which concludes there is limited evidence to support the use of some manual therapies for providing long-term relief of pain at myofascial trigger points [174]. Fifteen RCTs evaluating the effectiveness of manual therapy for the treatment of myofascial pain syndrome were included in the published systematic review [90,175-188]. Only two of the truly randomized trials assessed the effectiveness of manual therapy beyond the immediate post-treatment period [175,178]. One trial assessed the effectiveness of massage combined with other therapies, while the other trial assessed the effectiveness of self-treatment with ischemic compression.

#### Evidence-based clinical guidelines

None

#### Recent randomized clinical trials not included in above

None

#### Evidence Summary (See Figure 5)

◦ Inconclusive evidence in a favorable direction regarding the effectiveness of massage for the treatment of myofascial pain syndrome [174].

#### Other effective non-invasive physical treatments or patient education

Laser, acupuncture [174]

### Migraine Headache

#### Definition

Migraine headache is defined as recurrent/episodic moderate or severe headaches which are usually unilateral, pulsating, aggravated by routine physical activity, and are associated with either nausea, vomiting, photophobia, or phonophobia [189,190].

#### Diagnosis

Diagnosis of migraine headaches is made primarily from the patient's history and a negative neurological exam. Neuroimaging is only indicated in patients with a positive neurological exam or presence of a "red flag" [190].

### Evidence base for manual treatment

#### Systematic reviews (most recent)

Since 2004, two systematic reviews evaluated the benefit of manual therapy for migraine headache [191,192]. The reviews evaluated three RCTs on spinal manipulation [193-195]. Astin and Ernst [191] concluded that due to methodological limitations of the RCTs, it is unclear whether or not spinal manipulation is an effective treatment for headache disorders. In contrast, the conclusion from a Cochrane review [192] was that spinal manipulation is an effective option for the care of migraine headache. The conclusions of the two reviews differed in methodology for determining RCT quality and the strength of evidence. Astin and Ernst [191] evaluated study quality using a scale that is no longer recommended by the Cochrane Collaboration and did not apply evidence rules for their conclusions. The Cochrane review [192] used a pre-specified, detailed protocol for synthesizing the evidence from the quality, quantity, and results of RCTs.

#### Evidence-based clinical guidelines

The SIGN guidelines [190] for the diagnosis and management of headache in adults concludes the evidence of effectiveness for manual therapy is too limited to lead to a recommendation.

#### Recent randomized clinical trials not included in above

Lawler and Cameron [196] found that massage therapy significantly reduced migraine frequency in the short term compared to filling out a diary with no other treatment. This study had a high risk of bias.

#### Evidence Summary (See Figure 5)

◦ Moderate quality evidence that spinal manipulation has an effectiveness similar to a first-line prophylactic prescription medication (amitriptyline) for the prophylactic treatment of migraine [195].

◦ Inconclusive evidence in a favorable direction comparing spinal manipulation to sham interferential [194].

◦ Inconclusive evidence in a favorable direction regarding the use of massage therapy alone [196].

#### Other effective non-invasive physical treatments or patient education

Trigger avoidance, stress management, acupuncture, biofeedback [190,197,198]

### Tension- Type Headache

#### Definition

Tension-type headache is defined as a headache that is pressing/tightening in quality, mild/moderate in intensity, bilateral in location, and does not worsen with routine physical activity [189,190].

#### Diagnosis

Diagnosis of tension-type headaches is made primarily from the patient's history and a negative neurological exam [190]. Neuroimaging is only indicated in patients with a positive neurological exam or presence of a "red flag" [190].

### Evidence base for manual treatment

#### Systematic reviews (most recent)

Since 2002, five systematic reviews evaluated the benefit of manual therapy for tension-type headache [191,192,199-201]. Eleven RCTs were included in the published systematic reviews [202-212]. Three of the RCTs assessed the effectiveness of spinal manipulation [202,206,210], six of the trials evaluated the use of combined therapies including a form of manual therapy [203,207-209,211,212], one trial evaluated a craniosacral technique [204], and the remaining trial compared connective tissue manipulation to mobilization [205]. The reviews generally conclude there is insufficient evidence to draw inference on the effectiveness of manual therapy in the treatment of tension-type headache. An exception is the Cochrane review [192] which found that some inference regarding spinal manipulation could be made from two trials with low risk of bias. One trial [202] showed that for the prophylactic treatment of chronic tension-type headache, amitriptyline (an effective drug) is more effective than spinal manipulation during treatment. However, spinal manipulation is superior in the short term after cessation of both treatments, but this could be due to a rebound effect of the medication withdrawal. The other trial [203] showed that spinal manipulation in addition to massage is no more effective than massage alone for the treatment of episodic tension-type headache.

#### Evidence-based clinical guidelines

The SIGN guideline [190] for the diagnosis and management of headache in adults draws no conclusions.

#### Recent randomized clinical trials not included in above

Anderson and Seniscal [213] found that participants receiving osteopathic manipulation in addition to relaxation therapy had significant improvement in headache frequency compared to relaxation therapy alone. This study had a moderate risk of bias.

#### Evidence Summary (See Figure 5)

◦ Moderate quality evidence that spinal manipulation in addition to massage is no more effective than massage alone for the treatment of episodic tension-type headache [192,203].

◦ Inconclusive evidence in an unclear direction regarding the use of spinal manipulation alone or in combination with therapies other than massage for most forms of tension-type headache [191,192,199-202].

#### Other effective non-invasive physical treatments or patient education

Acupuncture, biofeedback [198,214]

### Cervicogenic Headache

#### Definition

Cervicogenic headache is defined as unilateral or bilateral pain localized to the neck and occipital region which may project to regions on the head and/or face. Head pain is precipitated by neck movement, sustained awkward head positioning, or external pressure over the upper cervical or occipital region on the symptomatic side [189,190,215].

#### Diagnosis

Diagnosis of cervicogenic headaches is made primarily from the patient's history and a negative neurological exam. Neuroimaging is only indicated in patients with a positive neurological exam or presence of a "red flag" [190].

### Evidence base for manual treatment

#### Systematic reviews (most recent)

Since 2002, four systematic reviews have been published on manual therapy for cervicogenic headache [55,191,192,216]. The reviews made inference based on six RCTs that evaluated a range of manual therapy treatments including spinal manipulation [217-222], mobilization [217,220], and friction massage [220,222]. Astin and Ernst [191] concluded that due to methodological limitations of the RCTs, it is unclear whether or not spinal manipulation is an effective treatment for headache disorders. In contrast, a Cochrane review [192]concluded that spinal manipulation is an effective option for the care of cervicogenic headache. The conclusions of the two reviews differed in methodology for determining RCT quality and the strength of evidence. Ernst [191] evaluated study quality using a scale that is no longer recommended by the Cochrane Collaboration and did not apply evidence rules for their conclusions. The Cochrane review [192] used a pre-specified, detailed protocol for synthesizing the evidence from the quality, quantity, and results of RCTs.

#### Evidence-based clinical guidelines

The SIGN guidelines [190] for the diagnosis and management of headache in adults concluded spinal manipulation should be considered in patients with cervicogenic headache.

#### Recent randomized clinical trials not included in above

Hall et al [223] evaluated the efficacy of apophyseal glide of the upper cervical region in comparison to a sham control. They found a large clinically important and statistically significant advantage of the intervention over sham for pain intensity. The study had a low risk of bias.

#### Evidence Summary (See Figure 5)

◦ Moderate quality evidence that spinal manipulation is more effective than placebo manipulation, friction massage, and no treatment [192].

◦ Moderate quality evidence that spinal manipulation is similar in effectiveness to exercise [220].

◦ Moderate quality evidence that self-mobilizing natural apophyseal glides are more effective than placebo [223].

◦ Inclusive evidence that deep friction massage with trigger point therapy is inferior to spinal manipulation [221].

◦ Inconclusive evidence in an unclear direction for the use of mobilization [192].

#### Other effective non-invasive physical treatments or patient education

Neck exercises [192]

### Miscellaneous Headache

#### Definition

Headaches not classified as tension-type, migraine, or cervicogenic in nature according to the International Headache Society's 2004 diagnostic criteria [189].

### Evidence base for manual treatment

#### Systematic reviews (most recent)

One systematic review (2004) evaluated the benefit of manual therapy for other types of chronic headache [192]. One RCT evaluating the use of mobilization for post-traumatic (post-concussive) headache was included [224]. The review found the evidence to be inconclusive.

#### Evidence-based clinical guidelines

None

#### Recent randomized clinical trials not included in above

None

#### Evidence Summary (See Figure 5)

◦ Inconclusive evidence in a favorable direction regarding mobilization for post-traumatic headache [224].

#### Other effective non-invasive physical treatments or patient education

None

### Asthma

#### Definition

Asthma is a common, complex chronic disorder of the airways that is characterized by variable and recurring symptoms, airflow obstruction, bronchial hyperresponsiveness, and an underlying inflammation [225].

#### Diagnosis

The diagnosis is made through the combination of the patient's history, upper respiratory physical exam, and pulmonary function testing (spirometry). Patient administered peak flow measurement is often used to monitor effects of treatment [225,226].

### Evidence base for manual treatment

#### Systematic reviews

Since 2002, four systematic reviews, one a Cochrane review, on manual therapy for asthma have been published [227-230]. Of the total of five RCTs on the effectiveness of manual therapy [231-235] available from the searched literature, two investigated chiropractic spinal manipulation for chronic asthma, one in adults [231] and the other in children [232]. Two trials assessed the effectiveness on chronic asthma in children, one examined osteopathic manipulative/manual therapy [233], and the other massage [234]. The fifth trial evaluated the effect of foot manual reflexology for change in asthma symptoms and lung function in adults [235]. The four systematic reviews collectively concluded that the evidence indicates that none of the manual therapy approaches have been shown to be superior to a suitable sham manual control on reducing severity and improving lung function but that clinically important improvements occur over time during both active and sham treatment.

#### Evidence-based clinical guidelines

The asthma guidelines by The US National Heart, Lung, and Blood Institutes [225] and by The British Thoracic Society [226] both conclude that there is insufficient evidence to recommend the use of chiropractic or related manual techniques in the treatment of asthma.

#### Recent randomized clinical trials not included in above

None

#### Evidence Summary (See Figures 6 &7)

◦ There is moderate quality evidence that spinal manipulation is not effective (similar to sham manipulation) for the treatment of asthma in children and adults on lung function and symptom severity [227,228].

◦ There is inconclusive evidence in a non-favorable direction regarding the effectiveness of foot manual reflexology for change in asthma symptoms and lung function in adults [235].

◦ There is inconclusive evidence in a favorable direction regarding the effectiveness of osteopathic manipulative treatment for change in asthma symptoms and lung function in children [233].

◦ There is inconclusive evidence in an unclear direction regarding the effectiveness of massage for change in asthma symptoms and lung function in children [234].

#### Other effective non-invasive physical treatments or patient education

Education and advice on self-management, maintaining normal activity levels, control of environmental factors and smoking cessation [225,226]

### Pneumonia

#### Definition

Pneumonia is defined as an acute inflammation of the lungs caused by infection [236,237].

#### Diagnosis

Diagnosis of pneumonia relies primarily on chest radiography in conjunction with the patient's history, examination, and laboratory findings [236,237].

### Evidence base for manual treatment

#### Systematic reviews (most recent)

Since 2007, one systematic review evaluating the benefit of manual therapy for pneumonia has been published [230]. One RCT evaluating the effectiveness of manual therapy for the treatment of pneumonia was included in the published systematic review [238]. The included trial assessed the effectiveness of osteopathic spinal manipulation for acute pneumonia in hospitalized elderly adults. The review concluded there is promising evidence for the potential benefit of manual procedures for hospitalized elderly patients with pneumonia. Our risk of bias assessment places this trial in the moderate risk of bias category.

#### Evidence-based clinical guidelines

None addressing the use of manual therapy

#### Randomized clinical trials not included in above

None

#### Evidence Summary (See Figure 6)

◦ There is inconclusive evidence in a favorable direction regarding the effectiveness of osteopathic manual treatment for the treatment of acute pneumonia in elderly hospitalized patients [238].

#### Other effective non-invasive physical treatments or patient education

Cases of pneumonia that are of public health concern should be reported immediately to the local health department. Respiratory hygiene measures, including the use of hand hygiene and masks or tissues for patients with cough, should be used in outpatient settings as a means to reduce the spread of respiratory infections [236,237].

### Vertigo

#### Definition

Vertigo is defined as a false sensation of movement of the self or the environment. Vertigo is a sensation and not necessarily a diagnosis as there are multiple underlying pathologies responsible for vertigo [239,240].

#### Diagnosis

Diagnosis of vertigo relies primarily on the patient's history and clinical examination. Potential causes of vertigo include both pathological disorders such as vertebrobasilar insufficiency or central nervous system lesions as well as more benign causes such as cervicogenic vertigo or benign paroxysmal positional vertigo [239].

### Evidence base for manual treatment

#### Systematic reviews (most recent)

Since 2004, two systematic reviews evaluating the benefit of manual therapy for vertigo have been published [230,240]. One RCT evaluating the effectiveness of mobilization and soft-tissue massage for the treatment of cervicogenic vertigo was included in both published systematic reviews [241]. One review concluded limited evidence of effectiveness [240]. The other concluded effectiveness, but the inference was on the inclusion of other types of evidence [230].

#### Evidence-based clinical guidelines

None addressing the use of manual therapy

#### Recent randomized clinical trials not included in above

Reid et al [242] compared sustained natural apophyseal glides (SNAGs), delivered manually by a therapist, to detuned laser treatment for the treatment of cervicogenic dizziness. Patients receiving SNAGs reported less dizziness, disability and cervical pain after six weeks, but not at 12 weeks. This study had a low risk of bias.

#### Evidence Summary (See Figure 5)

◦ Moderate quality evidence that manual treatment (specifically sustained natural apophyseal glides) is an effective treatment for cervicogenic dizziness, at least in the short term [242].

#### Other effective non-invasive physical treatments or patient education

Particle repositioning maneuvers for benign paroxysmal positional vertigo, vestibular rehabilitation [239,243]

### Infantile Colic

#### Definition

Colic is a poorly defined condition characterized by excessive, uncontrollable crying in infants.

#### Diagnosis

The diagnosis of colic is based solely on the patient's history and the absence of other explanations for the excessive crying. The "rule of threes" is the most common criteria used in making a diagnosis of colic. The rule of three's is defined as an otherwise healthy and well fed infant with paroxysms of crying and fussing lasting for a total of three hours a day and occurring more than three days a week for at least three weeks [244,245].

### Evidence base for manual treatment

#### Systematic reviews (most recent)

Since 2003, six systematic reviews evaluating the benefit of manual therapy for infantile colic have been published [230,245-249]. Two of the systematic reviews evaluated the effectiveness of manual therapy for non-musculoskeletal [247] and pediatric [248] conditions as a whole but fail to draw specific conclusions regarding the use of manual therapy for infantile colic. Of the eight RCTs evaluating the effectiveness of manual therapy for the treatment of colic, five were included in the published systematic reviews [250-254]. All five of the trials assessed the effectiveness of chiropractic spinal manipulation for infantile colic. All four systematic reviews concluded there is no evidence manual therapy is more effective than sham therapy for the treatment of colic.

#### Evidence-based clinical guidelines

No clinical guidelines located

#### Randomized clinical trials not included in above

Hayden et al [255] found cranial osteopathy was more effective than no treatment for crying duration. This study had a high risk of bias

Huhtala et al [256] found no difference between groups treated with massage therapy or given a crib vibrator for crying duration. This study had a high risk of bias.

Arikan et al [257] found all four interventions (massage, sucrose solution, herbal tea, hydrolysed formula) showed improvement compared to a no treatment control group. This study had a moderate risk of bias.

#### Evidence Summary (See Figure 7)

◦ Moderate quality evidence that spinal manipulation is no more effective than sham spinal manipulation for the treatment of infantile colic [254].

◦ Inconclusive evidence in a favorable direction regarding the effectiveness of cranial osteopathic manual treatment and massage for the treatment of infantile colic [255,257].

#### Other effective non-invasive physical treatments or patient education

Reduce stimulation, herbal tea, and trial of hypoallergenic formula milk [258,259]

### Nocturnal Enuresis

#### Definition

Nocturnal enuresis is defined as the involuntary loss of urine at night, in the absence of organic disease, at an age when a child could reasonably be expected to be dry (typically at the age of five) [260].

#### Diagnosis

The diagnosis of nocturnal enuresis is derived mainly from the patient's history given the absence of other organic causes including congenital or acquired defects of the central nervous system. Psychological factors can be contributory in some children requiring proper assessment and treatment [261].

### Evidence base for manual treatment

#### Systematic reviews (most recent)

Since 2005, two systematic reviews, one a Cochrane review, evaluating the benefit of manual therapy for nocturnal enuresis were published [230,262]. The systematic reviews included a total of two randomized clinical trials [263,264]. Both of the included trials examined the use of spinal manipulation for nocturnal enuresis. Both reviews concluded there is insufficient evidence to make conclusions about the effectiveness of spinal manipulation for the treatment of enuresis.

#### Evidence-based clinical guidelines

None addressing manual therapy as a treatment option

#### Randomized clinical trials not included in above

None

#### Evidence Summary (See Figure 7)

◦ Inconclusive evidence in a favorable direction regarding the effectiveness of chiropractic care for the treatment of enuresis [230,262].

#### Other effective non-invasive physical treatments or patient education

Education, simple behavioral interventions, and alarm treatment [265]

### Otitis Media

#### Definition

Otitis media is characterized by middle ear inflammation which can exist in an acute or chronic state and can occur with or without symptoms [266].

#### Diagnosis

Diagnosis of otitis media relies on otoscopic signs and symptoms consistent with a purulent middle ear effusion in association with systemic signs of illness [266].

### Evidence base for manual treatment

#### Systematic reviews (most recent)

Hawk et al [230] found promising evidence for the potential benefit of spinal manipulation/mobilization procedures for children with otitis media. This was based on one trial [267]. Two other reviews specifically addressed spinal manipulation by chiropractors for non-musculoskeletal [247] and pediatric [248] conditions. Both found insufficient evidence to comment on manual treatment effectiveness or ineffectiveness for otitis media.

#### Evidence-based clinical guidelines

The American Academy of Pediatrics 2004 guidelines on the diagnosis and management of acute otitis media [268] concluded no recommendation for complementary and alternative medicine for the treatment of acute otitis media can be made due to limited data.

#### Recent randomized clinical trials not included in above

Wahl et al investigated the efficacy of osteopathic manipulative treatment with and without Echinacea compared to sham and placebo for the treatment of otitis media [269]. The study found that a regimen of up to five osteopathic manipulative treatments does not significantly decrease the risk of acute otitis media episodes. This study had a high risk of bias.

#### Evidence Summary (See Figure 7)

◦ Inconclusive evidence in an unclear direction regarding the effectiveness of osteopathic manipulative therapy for otitis media [267,269].

#### Other effective non-invasive physical treatments or patient education

Patient education and "watch and wait" approach for 72 hours for acute otitis media [266,268]

### Hypertension

#### Definition

Hypertension is defined as the sustained elevation of systolic blood pressure over 140 mmHg, diastolic blood pressure over 90 mm Hg, or both [270,271].

#### Diagnosis

Diagnosis of hypertension is made by the physical exam, specifically sphygmomanometry. The patient's history, clinical exam and laboratory tests help identify potential etiologies [270,271].

### Evidence base for manual treatment

#### Systematic reviews (most recent)

Since 2007, one systematic review evaluating the benefit of manual therapy for hypertension has been published (Hawk et al) [230]. Two RCTs evaluating the effectiveness of manual therapy for the treatment of stage I hypertension were included in this systematic review [272,273]. One of the included trials evaluated the use of spinal manipulation [272] and the other evaluated the use of instrument assisted spinal manipulation [273]. The review found no evidence of effectiveness for spinal manipulation.

#### Evidence-based clinical guidelines

None addressing the use of manual therapy

#### Recent randomized clinical trials not included in above

A study by Bakris et al [274] found NUCCA upper cervical manipulation to be more effective than sham manipulation in lowering blood pressure in patients with Stage I hypertension. This study had a high risk of bias.

#### Evidence Summary (See Figure 6)

◦ Moderate quality evidence that diversified spinal manipulation is not effective when added to a diet in the treatment of stage I hypertension [272].

◦ Inconclusive evidence in a favorable direction regarding upper cervical NUCCA manipulation for stage I hypertension [274].

◦ Inconclusive evidence in an unclear direction regarding instrument assisted spinal manipulation for hypertension [273].

#### Other effective non-invasive physical treatments or patient education

Advice on lifestyle interventions including diet, exercise, moderate alcohol consumption and smoking cessation [270,271]

Relaxation therapies including biofeedback, meditation, or muscle relaxation [271]

### Dysmenorrhea

#### Definition

Dysmenorrhea is defined as painful menstrual cramps of uterine origin. Dysmenorrhea is grouped into two categories, primary and secondary dysmenorrhea. Secondary dysmenorrhea is painful menstruation associated with a pelvic pathology like endometriosis, while primary dysmenorrhea is painful menstruation in the absence of pelvic disease [275].

#### Diagnosis

Diagnosis of primary dysmenorrhea is made from the patient's history. Diagnosis of secondary dysmenorrhea requires further investigation including a pelvic exam and potential ultrasound or laparoscopy [275].

### Evidence base for manual treatment

#### Systematic reviews (most recent)

We identified two systematic reviews evaluating the benefit of manual therapy for dysmenorrhea [230,276]. Five studies evaluating the effectiveness of manual therapy for the treatment of dysmenorrhea were included in the systematic reviews [277-281]. Four of the included trials examined the use of spinal manipulation [278-281] and one examined the use of osteopathic manipulative techniques [277]. Based on these trials, the Cochrane review by Proctor et al concluded there is no evidence to suggest that spinal manipulation is effective in the treatment of primary and secondary dysmenorrhea [276]. The review by Hawk et al concluded the evidence was equivocal regarding chiropractic care for dysmenorrhea [230].

#### Evidence-based clinical guidelines

We identified consensus guidelines from the Society of Obstetricians and Gynecologists of Canada (SOGC) published in 2005 which included an assessment of manual treatment for primary dysmenorrhea. The authors concluded there is no evidence to support spinal manipulation as an effective treatment for primary dysmenorrhea [275].

#### Recent randomized clinical trials not included in above

None

#### Evidence Summary (See Figure 7)

◦ Moderate quality evidence that spinal manipulation is no more effective than sham manipulation in the treatment of primary dysmenorrhea [276,281].

#### Other effective non-invasive physical treatments or patient education

High frequency TENS [275]

### Premenstrual Syndrome

#### Definition

Premenstrual syndrome is defined as distressing physical, behavioral, and psychological symptoms, in the absence of organic or underlying psychiatric disease, which regularly recurs during the luteal phase of the menstrual cycle and disappears or significantly regresses by the end of menstruation and is associated with impairment in daily functioning and/or relationships [282,283].

#### Diagnosis

Diagnosis of premenstrual syndrome is made through patient history and the use of a patient diary over two menstrual cycles [282,283].

### Evidence base for manual treatment

#### Systematic reviews (most recent)

Since 2007, three systematic reviews evaluating the benefit of manual therapy for premenstrual syndrome have been published [230,284,285]. Three RCTs evaluating the effectiveness of manual therapy for the treatment of premenstrual syndrome were included in the reviews [286-288]. The included trials examined different forms of manual therapy including spinal manipulation [286], massage therapy [287], and reflexology [288]. Overall, the reviews concluded that the evidence is "not promising" [284], "equivocal" [230], and that high quality studies are needed to draw firm conclusions [284,285].

#### Evidence-based clinical guidelines

None discussing manual therapy

#### Recent randomized clinical trials not included in above

None

#### Evidence Summary (See Figure 7)

◦ Inconclusive evidence in a favorable direction regarding the effectiveness of reflexology and massage therapy for the treatment of premenstrual syndrome [230].

◦ Inconclusive evidence in an unclear direction regarding the effectiveness of spinal manipulation for the treatment of premenstrual syndrome [230].

#### Other effective non-invasive physical treatments or patient education

Cognitive behavioral therapy [282]

## Discussion

### Making claims

There are two important questions underlying the medical and media debate surrounding the scope of chiropractic care and claims regarding its effectiveness particularly for non-musculoskeletal conditions: 1) should health professionals be permitted to use generally safe but as yet unproven methods? 2) What claims, if any, can and should be made with respect to the potential value of unproven treatments?

In response to the first question, a reasonable answer is "yes" given that professionals operate within the context of EBH, where it is acknowledged what is known today, might change tomorrow. It requires flexibility born of intellectual honesty that recognizes one's current clinical practices may not *really *be in the best interests of the patient and as better evidence emerges, clinicians are obligated to change. Further, where evidence is absent, they are open to promoting the development of new knowledge that expands understanding of appropriate health care delivery.

In response to the second question, no claims of efficacy/effectiveness should be made for which there isn't sufficient evidence. Unsubstantiated claims can be dangerous to patient health [289]. We maintain the best evidence for efficacy/effectiveness that meets society's standards comes from well-designed RCTs. While other study designs and clinical observations do offer insight into the plausibility and potential value of treatments, the concepts of plausibility and evidence of efficacy/effectiveness should not be confused when making claims.

### Clinical Experience versus Clinical effectiveness

Why is it that the results of RCTs often do not confirm the results observed in clinical practice? There are several reasons. One of the problems is that both the provider and the patient are likely to interpret any improvement as being solely a result of the intervention being provided. However this is seldom the case. First, the natural history of the disorder (for example. acute LBP) is expected to partially or completely resolve by itself regardless of treatment. Second, the phenomenon of regression to the mean often accounts for some of the observed improvement in the condition. Regression to the mean is a statistical phenomenon associated with the fact that patients often present to the clinic or in clinical trials at a time where they have relatively high scores on severity outcome measures. If measured repeatedly before the commencement of treatment the severity scores usually regress towards lower more normal average values [290].

Additionally, there is substantial evidence to show that the ritual of the patient practitioner interaction has a therapeutic effect in itself separate from any specific effects of the treatment applied. This phenomenon is termed contextual effects [1,291]. The contextual or, as it is often called, non-specific effect of the therapeutic encounter can be quite different depending on the type of provider, the explanation or diagnosis given [292], the provider's enthusiasm, and the patient's expectations [293-298]. Some researchers have suggested that relying on evidence from RCTs and systematic reviews of RCTs is not adequate to determine whether a treatment is effective or not. The main issue, they contend, is that the intervention when studied in RCTs is too highly protocolized and does not reflect what is going on in clinical practice [230]. They advocate a whole systems research approach that more accurately represents the entire clinical encounter. When using this perspective and systematically synthesizing the literature regarding chiropractic treatment of non-musculoskeletal conditions, also reviewed in this report, they conclude, for example that chiropractic is beneficial to patients with asthma and to children with infantile colic [230]. This conclusion is at odds with the evidence summaries found in this report. We submit that whole systems research approach in this instance is clouding the interpretation of the literature regarding effectiveness as it relates to making claims, and incorrectly giving the consumer the impression that chiropractic care shows effectiveness over and above the contextual effects as it relates to the two examples above.

In a placebo-controlled RCT the question is: does the treatment provided have a specific effect over and above the contextual or non-specific effects. The result of such a trial may show that there is no important difference between the active intervention and the sham intervention. However, the patients may exhibit clinically important changes from baseline in both groups and thus the outcome would be consistent with what clinicians observe in their practice. An example of this is the results of the pragmatic placebo controlled RCT on chiropractic co-management of chronic asthma in adults (care delivered by experienced chiropractors consistent with normal clinical practice), which showed that patients improved equally during both the active and the sham intervention phases of the trial [231].

### The Pieces of The Evidence-Based Healthcare Puzzle

It is essential to recognize what each piece of the EBH puzzle offers. Patient values and preferences do not provide sound evidence of a treatment's effectiveness and may be misleading. A patient can be satisfied with a treatment, but it still may not be effective. The clinician's observations, if well documented, can attest to patient improvement while under care and encourage perception of a treatment's clinical plausibility. However, the narrow focus of attention under non-systematic observations common to practice experience tends to obscure other factors influencing case outcome. Similarly, EBH can be flawed, not because it fails to be scientific, but because-like all sciences-it imports the biases of researchers and clinicians [299]. Well-performed clinical research however, does provide evidence for claims that a treatment is effective when the results are consistently applied to relevant patients. This is because of its reliance on methods for systematic observation and efforts to minimize bias.

Other authors' work has been used to argue that a range of study types should be included when evaluating a treatment's efficacy/effectiveness (case series, etc.) [230,300]. We maintain the best evidence that rises to societal standards to support claims of efficacy/effectiveness comes from well-designed RCTs. This is largely due to the powerful effect of successful randomization and design factors intended to minimize bias (all which help ensure that the results are due to the intervention and not some other known or unknown factor). Other evidence may be useful to inform treatment options when conditions for individual patients are not consistent with the best evidence or when better evidence is unavailable [11]. Other types of research are more appropriate for answering related questions including, but not limited to, safety or mechanistic plausibility. This can lead to the refinement of interventions, inform the design of clinical trials, and aid in the interpretation of clinical observations. Similarly, clinical data from epidemiological studies, case reports, and case series can suggest that a treatment is *clinically plausible*. That is, clinical observations demonstrate that *it is possible *that an intervention is effective. However, a gain in plausibility, biological or clinical, does NOT constitute proof of a treatment's efficacy in human populations. Conversely lack of proof (as demonstrated through well performed randomized clinical trials) does not exclude plausibility [301,302].

Research on systematic reviews have taught us that individual studies can often lead to a conclusion very different from that of a systematic analysis of all available studies [3]. Moreover, the scientific process is a systematic means of self-correcting investigations that classically begin with observations and hypotheses that support plausibility and/or mechanisms. Ideally, these precede and inform the conduct of RCTs under conditions most likely to yield clear results, often referred to as efficacy studies. Separately, studies that emulate general practice conditions may be used to develop an understanding of effectiveness. Historically, the modern investigation of manual treatment methods represents an aberration in this process. With the advent of social support and funding for research at the end of the 20^th ^Century, there was an underlying presumption that the long-term practice of these methods provided a sound clinical wisdom on which to ground RCTs, bypassing mechanistic studies. The early emphasis on clinical trials has illuminated the gaps in understanding of appropriate indications for treatment, dosage and duration of care, consistency of treatment application, and the appropriate outcome measures to monitor results [11]. In response, funding agencies in North America have renewed research emphasis on the potential mechanisms of effect [303]. Data from this work is expected to inform future clinical research questions, and subsequently lead to well-grounded studies that are likely to yield more complete evidence regarding appropriate and effective care.

### Safety of Manual Treatment

Choosing an intervention should always be tempered by the risk of adverse events or harm. Adverse events associated with manual treatment can be classified into two categories: 1) benign, minor or non-serious and 2) serious. Generally those that are benign are transient, mild to moderate in intensity, have little effect on activities, and are short lasting. Most commonly, these involve pain or discomfort to the musculoskeletal system. Less commonly, nausea, dizziness or tiredness are reported. Serious adverse events are disabling, require hospitalization and may be life-threatening. The most documented and discussed serious adverse event associated with spinal manipulation (specifically to the cervical spine) is vertebrobasilar artery (VBA) stroke [304,305]. Less commonly reported are serious adverse events associated with lumbar spine manipulation, including lumbar disc herniation and cauda equina syndrome [304].

Estimates of serious adverse events as a result of spinal manipulation have been uncertain and varied. Much of the available evidence has been relatively poor due to challenges in establishing accurate risk estimates for rare events. Such estimates are best derived from sound population based studies, preferably those that are prospective in nature [304,306].

Estimates of VBA stroke subsequent to cervical spine manipulation range from one event in 200,000 treatments to one in several million [307,308]. In a subsequent landmark population-based study, Cassidy et al [309] revisited the issue using case-control and case-crossover designs to evaluate over 100 million person-years of data. The authors confirmed that VBA stroke is a very rare event in general. They stated, "We found no evidence of excess risk of VBA stroke associated with chiropractic care compared to primary care." They further concluded, "The increased risk of VBA stroke associated with chiropractic and PCP (primary care physician) visits is likely due to patients with headache and neck pain from VBA dissection seeking care before their stroke." In regards to benign adverse reactions, cervical spine manipulation has been shown to be associated with an increased risk when compared to mobilization [55,310,311].

Appropriately, the risk-benefit of cervical spine manipulation has been debated [304,305]. As anticipated, new research can change what is known about the benefit of manual treatment for neck pain. Currently, the evidence suggests that it has some benefit [55]. It has been suggested that the choice between mobilization and manipulation should be informed by patient preference [55].

Estimates of cervical or lumbar disc herniation are also uncertain, and are based on case studies and case series. It has been estimated that the risk of a serious adverse event, including lumbar disc herniation is approximately 1 per million patient visits [312]. Cauda equina syndrome is estimated to occur much less frequently, at 1 per several million visits [312-314].

### Safety of Manual Treatment in Children

The true incidence of serious adverse events in children as a result of spinal manipulation remains unknown. A systematic review published in 2007 identified 14 cases of direct adverse events involving neurologic or musculoskeletal events, nine of which were considered serious (eg. subarachnoid hemorrhage, paraplegia, etc.) [315]. Another 20 cases of indirect adverse events were identified (delayed diagnosis, inappropriate application of spinal manipulation for serious medical conditions). The review authors note that case reports and case series are a type of "passive" surveillance, and as such don't provide information regarding incidence. Further, this type of reporting of adverse events is recognized to underestimate true risk [315-317].

Importantly, the authors postulate that a possible reason for incorrect diagnosis (for example. delayed diagnosis, inappropriate treatment with spinal manipulation) is due to lack of sufficient pediatric training. They cite their own survey [318] which found that in a survey of 287 chiropractors and osteopaths, 78% reported one semester or less of formal pediatric education and 72% received no pediatric clinical training. We find this particularly noteworthy.

### Limitations of the Report Conclusions

The conclusions in this report regarding the strength of evidence of presence or absence of effectiveness are predicated on the rules chosen for which there are no absolute standards. Different evidence grading systems and rules regarding impact of study quality may lead to different conclusions. However, we have applied a synthesis methodology consistent with the latest recommendations from authoritative organizations involved in setting standards for evidence synthesis. Although we used a comprehensive literature search strategy we may not have identified all relevant RCTs, guidelines, and technology reports. Conditions for which this report concludes the evidence currently shows manual treatment to be effective or even ineffective, sometimes rests on a single RCT with adequate statistical power and low risk of bias. Additional high quality RCTs on the same topics have a substantial likelihood of changing the conclusions. Including only English language reviews and trials may be considered another limitation of this report leading to language bias; however, the impact of excluding non-English trials from meta-analyses and systematic reviews is conflicting [319,320], and the incidence of randomized trials published in non-English journals is declining [321]. Another potential limitation of this report is the lack of critical appraisal of the systematic reviews and clinical guidelines included in the report. Systematic reviews and clinical guidelines can differ widely in methodologic quality and risk of bias [322]. While critical appraisal of the included reviews and guidelines would be ideal, it was beyond the scope of the present report. When drawing conclusions about relative effectiveness of different forms of manual treatments it is acknowledged that it has usually not been possible to isolate or quantify the specific effects of the interventions from the non-specific (contextual) effect of patient-provider interaction [291]. It was beyond the scope of this report to assess the magnitude of the effectiveness of the different manual therapies relative to the therapies to which comparisons were made. However, if moderate or high quality evidence of effectiveness was established the therapy was interpreted as a viable treatment option, but not necessarily the most effective treatment available. We recognize that findings from studies using a nonrandomized design (for example. observational studies, cohort studies, prospective clinical series and case reports) can yield important preliminary evidence on potential mechanisms and plausibility of treatment effects. However, the primary purpose of this report is to summarize the results of studies designed to specifically address treatment efficacy and effectiveness from which claims of clinical utility, consistent with that literature, may be considered defensible. Therefore, the evidence base on the effects of care was restricted to RCTs.

## Conclusions

Spinal manipulation/mobilization is effective in adults for acute, subacute, and chronic low back pain; for migraine and cervicogenic headache; cervicogenic dizziness; and a number of upper and lower extremity joint conditions. Thoracic spinal manipulation/mobilization is effective for acute/subacute neck pain, and, when combined with exercise, cervical spinal/manipulation is effective for acute whiplash-associated disorders and for chronic neck pain. The evidence is inconclusive for cervical manipulation/mobilization alone for neck pain of any duration, and for any type of manipulation/mobilization for mid back pain, sciatica, tension-type headache, coccydynia, temporomandibular joint disorders, fibromyalgia, premenstrual syndrome, and pneumonia in older adults. Spinal manipulation is not effective for asthma and dysmenorrhea when compared to sham manipulation, or for Stage 1 hypertension when added to an antihypertensive diet. For children, the evidence is inconclusive regarding the effectiveness of spinal manipulation/mobilization for otitis media and enuresis, but shows it is not effective for infantile colic and for improving lung function in asthma when compared to sham manipulation.

The evidence regarding massage shows that for adults it is an effective treatment option for chronic LBP and chronic neck pain. The evidence is inconclusive for knee osteoarthritis, fibromyalgia, myofascial pain syndrome, migraine headache, and premenstrual syndrome. For children, the evidence is inconclusive for asthma and infantile colic.

## Competing interests

All authors are trained as doctors of chiropractic but are now full time professional researchers.

## Authors' contributions

GB was responsible for the methodology used to select and summarize the evidence, for organizing and participating in the analysis of the evidence and formulating conclusions and drafting and finalizing the report.

MH participated in analyzing the evidence and formulating conclusions for the majority of the musculoskeletal conditions and the different types of headache.

RE participated in analyzing the evidence and formulating conclusion for part of the musculoskeletal and non-musculoskeletal conditions and providing substantial input to the background and discussion sections.

BL was responsible for retrieving the research articles and providing draft summary statements for all conditions as well as participating in drafting and proof reading the manuscript.

JT was responsible for conceiving and drafting the section on translation of research into action and providing substantial input to the background and discussion sections. All authors have read and approved the final manuscript.

## Supplementary Material

Additional file 1 The literature search strategy. Click here for file

Additional file 2 Includes the criteria used for evaluating risk of bias from randomized controlled trials not included within systematic reviews, evidence based guidelines, or health technology assessments. Click here for file
