# Supplementary material for: Effectiveness of manual therapies: the UK evidence report
Source: Chiropr Osteopat. 2010 Feb 25;18:3. doi: 10.1186/1746-1340-18-3 (PMC2841070; doi:10.1186/1746-1340-18-3)
Supplement: Additional file 1 — The literature search strategy. [file 1746-1340-18-3-S1.DOC]

**MEDLINE search strategy**

The full MEDLINE search strategy is listed below and was adapted for use in the other electronic databases searched.

Database: Ovid MEDLINE(R) <1996 to September 2009>

Line 1 is an example of one condition. The following conditions were searched individually and exploded when relevant with the same strategy from line 2 on:

Asthma, colic, dysmenorrhea, enuresis, extremities or upper extremity or lower extremity, fibromyalgia headache disorders, hypertension, low back pain, mind-body therapies, neck pain, otitis media, phobic disorders, pneumonia, premenstrual syndrome, thoracic and spine, and vertigo.

--------------------------------------------------------------------------------

1 exp asthma/

2 Manipulation, Orthopedic/ or Manipulation, Chiropractic/ or Manipulation, Spinal/ or Manipulation, Osteopathic/

3 Chiropractic/

4 nonpharmacological interventions.mp.

5 noninvasive interventions.mp.

6 ((physical$ or manual) adj (therap$ or intervention$ or treat$ or rehab$)).mp. [mp=title, original title, abstract, name of substance word, subject heading word]

7 (exercise adj therapy).mp. [mp=title, original title, abstract, name of substance word, subject heading word]

8 6 or 4 or 3 or 7 or 2 or 5

9 Controlled Clinical Trial/

10 Randomized Controlled Trial/

11 Randomized Controlled Trials as Topic/

12 11 or 10 or 9

13 "Scientific Integrity Review"/ or "Review"/ or "Review Literature as Topic"/

14 systematic review.mp.

15 13 or 14

16 Evidence-Based Medicine/

17 Guideline/ or Practice Guideline

18 16 or 17 or 12 or 15

19 8 and 1

20 18 and 19

21 limit 20 to english language
