# Supplementary material for: Effectiveness of manual therapies: the UK evidence report
Source: Chiropr Osteopat. 2010 Feb 25;18:3. doi: 10.1186/1746-1340-18-3 (PMC2841070; doi:10.1186/1746-1340-18-3)
Supplement: Additional file 2 — Includes the criteria used for evaluating risk of bias from randomized controlled trials not included within systematic reviews, evidence based guidelines, or health technology assessments. [file 1746-1340-18-3-S2.DOC]

## The Cochrane Collaboration tool for assessing risk of bias and the rating of the bias for the purpose of this report

| **Domain** | **Description** | **Judgement** |
| --- | --- | --- |
| Random sequence generation. | Describe the method used to generate the allocation sequence in sufficient detail to allow an assessment of whether it should produce comparable groups. | Was the allocation sequence adequately generated? |
| Random allocation concealment. | Describe the method used to conceal the allocation sequence in sufficient detail to determine whether intervention allocations could have been foreseen in advance of, or during, enrolment. | Was allocation adequately concealed? |
| Blinding of participants, personnel and outcome assessors Assessments should be made for each main outcome (or class of outcomes). | Describe all measures used, if any, to blind study participants and personnel from knowledge of which intervention a participant received. Provide any information relating to whether the intended blinding was effective. | Was knowledge of the allocated intervention adequately prevented during the study? |
| Incomplete outcome data Assessments should be made for each main outcome (or class of outcomes). | Describe the completeness of outcome data for each main outcome, including attrition and exclusions from the analysis. State whether attrition and exclusions were reported, the numbers in each intervention group (compared with total randomized participants), reasons for attrition/exclusions where reported, and any re-inclusions in analyses performed by the review authors. | Were incomplete outcome data adequately addressed? |
| Selective outcome reporting. | State how the possibility of selective outcome reporting was examined by the review authors, and what was found. | Are reports of the study free of suggestion of selective outcome reporting? |
| Other sources of bias. | State any important concerns about bias not addressed in the other domains in the tool.  If particular questions/entries were pre-specified in the review’s protocol, responses should be provided for each question/entry. | Was the study apparently free of other problems that could put it at a high risk of bias? |

**Interpretation for the purpose of this report is guided by suggestions in the Cochrane Handbook** <http://www.cochrane.org/resources/handbook/>**:**

Prerequisite for low and moderate risk: no serious methodological or analysis flaws

Low risk: Minimum 4 positive and no negatives. (allows 2 or less unclear)

Moderate risk: Minimum 4 positive and maximum of 1 negative (allows 1 unclear) or 3 positive and 3 unclear.

High risk: everything below moderate and low risk.
